# Organic fertilizer-mediated assembly of rhizosphere core microbiota contributes to increased sugarcane productivity

**DOI:** 10.3389/fpls.2025.1689397

**Published:** 2026-01-30

**Authors:** Jun Deng, Zhongfu Zhang, Yutong Wang, Jing Ai, Yong Zhao, Bao Li, Hongjun Li, Yan Deng, Fusuo Zhang

**Affiliations:** 1 State Key Laboratory of Nutrient Use and Management, College of Resources and Environmental Sciences, National Academy of Agriculture Green Development, China Agricultural University, Beijing, China; 2 Sugarcane Research Institute, Yunnan Academy of Agricultural Sciences, Kaiyuan, China; 3 Gengma Dai and Wa Autonomous County Local Industry Development Service Center, Gengma, China; 4 College of Resources and Environment, Southwest University, Chongqing, China

**Keywords:** fertilizer, nutrition soil, rhizosphere, soil microbial, sugarcane productivity

## Abstract

Optimizing fertilization strategies to enhance crop yield remains a critical challenge in sustainable agriculture. In this study, two field experiments were conducted in Yunnan Province, China, to evaluate the effects of three fertilization regimes—no fertilization (CK), chemical fertilization (F), and combined chemical and organic fertilization (FM)—on sugarcane yield, soil biochemical properties, and rhizosphere bacterial communities. The results showed that the FM treatment significantly increased sugarcane yield at both sites compared to CK and F. This yield improvement was accompanied by increased soil nutrient availability, enhanced enzyme activities, and significant changes in microbial community structure. High-throughput 16S rRNA sequencing results indicated that the FM treatment selectively enriched specific taxa, such as Actinobacteriota and Proteobacteria, and promoted the development of a more modular and stable microbial co-occurrence network. Correlation analysis and structural equation modeling further revealed that the FM treatment improved sugarcane yield primarily through microbiome-driven pathways that indirectly enhanced soil biochemical functions. Overall, this study unveils the mechanistic links between integrated fertilization strategies, rhizosphere microbial network stability, and sugarcane yield, providing scientific evidence for sustainable fertilization management in sugarcane production systems.

## Introduction

The expanding global population and diminishing agricultural land are placing immense pressure on agricultural production (Hossain et al., 2020), and thus improving crop productivity (particularly on limited land resources) has become a key objective in global agricultural research (Guan et al., 2025). It is of significance to enhance agricultural production efficiency within a sustainable framework (Shi et al., 2023; Xie et al., 2023). Recent studies have demonstrated that soil microorganisms, particularly rhizosphere microbial communities, play a crucial role in plant growth, crop yield, and environmental adaptability (Luo et al., 2025). Rhizosphere microorganisms not only enhance nutrient uptake by plants and improve nutrient use efficiency, but also promote plant resilience under stress conditions such as drought and salinity by fostering the proliferation of beneficial microbes (Li et al., 2025). Therefore, effectively regulating and optimizing the structure of rhizosphere microbial communities to enhance soil ecological functions has emerged as a key strategy for promoting sustainable agricultural development by improving both crop productivity and soil health.

In crop production, fertilization serves as a fundamental strategy for providing essential nutrients to crops while simultaneously regulating the structure and function of soil microbial communities (Yousaf et al., 2017). Numerous studies have demonstrated that various types of fertilizers can impact soil nutrient availability directly, and influence the composition and activity of microbial communities indirectly by altering soil physicochemical properties (Dang et al., 2022). Chemical fertilizers are widely applied due to their rapid nutrient release, effectively meeting crop nutrient demands in a timely manner (Philippot et al., 2024). However, prolonged use of chemical fertilizers has been associated with adverse effects, including soil acidification, depletion of organic matter, and a reduction in microbial diversity, ultimately compromising soil health (Tripathi et al., 2020). In contrast, organic fertilizers provide organic matter and essential micronutrients, which improve soil structure and offer a stable, long-term nutrient source for microorganisms, thereby enhancing microbial activity. Nevertheless, the nutrient release from organic fertilizers is relatively slow, resulting in less immediate effects on crop growth compared to chemical fertilizers.

Considering the differences in nutrient release characteristics between chemical fertilizers and organic fertilizers, the combined application of both (FM) has attracted increasing attention as a promising fertilization strategy in recent years (Liu et al., 2021a). The FM strategy integrates the rapid nutrient supply provided by chemical fertilizers with the sustained input of organic matter from organic fertilizers, thereby improving the rhizosphere environment, promoting the proliferation of beneficial microorganisms, enhancing crop yield, and improving soil health (Yu et al., 2024). Consequently, integrating chemical and organic fertilizers to regulate rhizosphere microbial communities has become a critical focus in the advancement of sustainable agricultural practices (Yang et al., 2025). Previous studies have demonstrated that the FM treatments generally exert significantly greater effects on crop growth compared to the sole application of chemical or organic fertilizers (Cui et al., 2018). For instance, combined fertilization significantly enhanced soil organic matter, total nitrogen content, and maize yield in field trials (Wei et al., 2016). Similarly, FM application in rice-growing regions effectively modulated rhizosphere microbial community structure, facilitated the enrichment of beneficial microorganisms, and improved nitrogen use efficiency (Chen et al., 2022). However, whether such combined application could optimize the rhizosphere microbial co-occurrence network structure, enhance network stability, and ultimately increase sugarcane yield remains insufficiently explored in sugarcane cultivation systems.

Considering the research gap, the present study aims to systematically evaluate the effects of no fertilizer application (CK), chemical fertilizer (F), and combined application of chemical and organic fertilizers (FM) on the composition of rhizosphere microbial communities, microbial network structure, and sugarcane yield through field experiments. Specifically, the objectives of this study are: (1) to elucidate the regulatory effects of different fertilization treatments on soil physicochemical properties and enzyme activities; (2) to construct a microbial co-occurrence network model to identify keystone taxa with potential regulatory influence on sugarcane yield; and (3) to investigate the impacts of FM treatment on the stability and dynamic changes of rhizosphere microbial networks. The findings will provide a scientific basis for optimizing fertilization strategy in sugarcane-growing areas, enhancing both sugarcane productivity and soil health, and offer theoretical insights into the response mechanisms of soil microbial networks to fertilization management.

## Materials and methods

### Experimental site and treatment design

The field experiments were conducted in Gengma County, Lincang City, one representative sugarcane-producing region in Yunnan Province, China. Trials were established at two field sites: Huaqiao (23.54874°N, 99.44432°E) with an area of 5,280 m^2^ (35.2 m × 150 m) and Bankang (23.53481°N, 99.46026°E) with an area of 14,400 m^2^ (48 m × 300 m). In mid-March of 2023, sugarcane variety ‘Yuetang 93-159’ was planted at the Huaqiao site with a row spacing of 1.1 m, while ‘Yunzhe 08-1609’ was grown at the Bankang site with a row spacing of 1.2 m. The plant-cane was harvested in mid-February of 2024, and then the first ratoon-cane season started. A randomized complete block design was adopted at each site with three fertilization treatments: no fertilizer (control, CK), chemical fertilizer only (F), and a combination of chemical and organic fertilizers (FM). The F treatment included two fertilizer schemes: a new compound fertilizer (N:P:K = 16-7-13, 36% of total nutrients) applied at 1500 kg ha^-1^, and a traditional compound fertilizer (N:P:K = 26-12-6, 44% of total nutrients) applied at 1200 kg ha^-1^. The FM treatment also included two schemes: a new organic-inorganic compound fertilizer (N:P:K = 17-7-9, 33% of total nutrients, 15% of organic content) applied at 1650 kg ha^-1^, and a combination of the new compound fertilizer (N:P:K = 16-7-13, 36% of total nutrients, 1200 kg ha^-1^) with a bio-organic fertilizer (5% of total nutrients, 45% of organic content, 2×10^8^ CFU/g of viable bacteria count, 1800 kg ha^-1^). Field plot was replicated four times for CK and eight times for both F and FM at each site. One-time fertilization as basal dressing was applied in each cropping season: all fertilizers were applied in the planting furrow before planting (15th-17th March, 2023) for the plant-cane, and along the planting row after plant-cane harvest (23rd-25th February, 2024) for the ratoon-cane. After the fertilizer application, film mulching was applied to all the treatments. The present study from the first ratoon crop (2024), with the plant-cane having been planted and harvested in 2023.

### Measurement of sugarcane stem number and yield

At the sugarcane maturity stage (Mid-December, 2024) harvest was conducted at both sites. For each plot, canes of two rows (10 m long) in the central area were firstly counted to determine stem number (stems/ha), and then manually harvested and weighed to measure yield per hectare (t/ha).

### Soil sampling and physicochemical properties analysis

**Figure 1:**
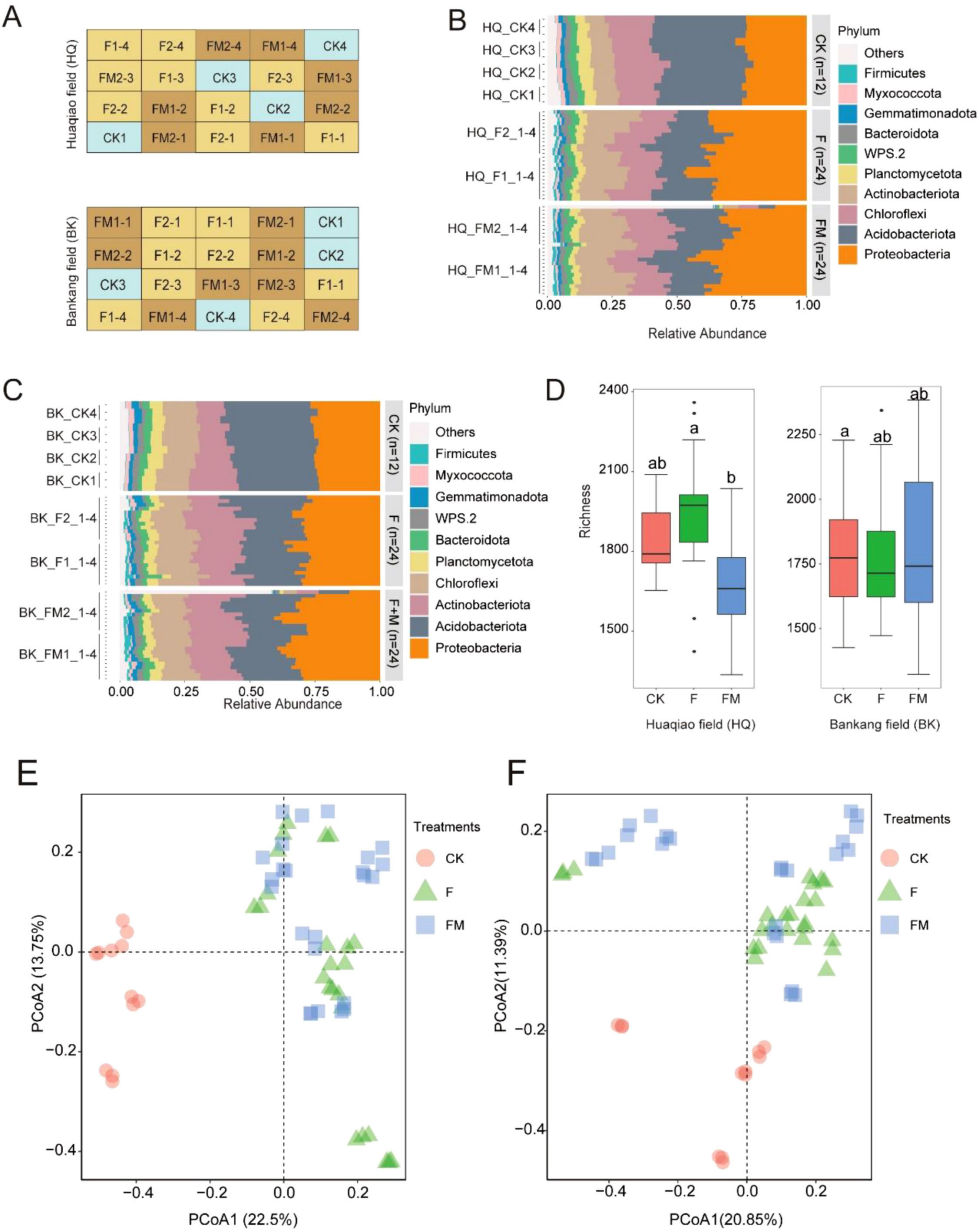
Changes in microbial diversity and community composition under different fertilization treatments. **(A)** Diagram of the original sampling sites in the Huaqiao (HQ) and Bankang (BK) fields, with three different fertilizer application types. Each sample is labeled according to the treatment and replicate (e.g., HQ_F1-1). **(B, C)** The relative abundance of microbial phyla under different fertilization treatments in Huaqiao and Bankang fields, with each microbial phylum represented by a different color. **(D)** Boxplots of microbial richness under different fertilization treatments in both fields. **(E, F)** Unconstrained PCoA (for principal coordinates PCo1 and PCo2) with Bray–Curtis distance showing dissimilarity for the Huaqiao and Bankang fields, respectively (*p* < 0.001, permutational multivariate analysis of variance (PERMANOVA) by Adonis). CK, no fertilizer application; F, only chemical fertilizer application; FM, combined chemical and organic fertilizers application.

Rhizosphere soil samples were collected at the elongation stage of sugarcane in mid-July of 2024 in each plot (**Figure 1A**). Nine representative plants per plot were randomly selected, avoiding plants near the plot borders. Entire root systems were carefully excavated; loosely attached soil was shaken off, and rhizosphere soil adhering tightly to roots was collected, the sampling depth was 0–30 cm. These samples were pooled, passed through a 2 mm sieve, placed in 5 mL sterile centrifuge tubes, and stored in liquid nitrogen for total DNA extraction. In parallel, bulk soil (approximately 1500 g) was collected, homogenized, and split into two portions: ~1000 g for soil physicochemical analysis and ~500 g for soil enzyme activity assays. Soil physicochemical parameters included pH (electrode method), soil organic matter (SOM; potassium dichromate oxidation with external heating method), total nitrogen (TN; semi-micro Kjeldahl method), total phosphorus (TP; sodium hydroxide extraction and spectrophotometry method), total potassium (TK; sodium hydroxide extraction and flame photometry method), available nitrogen (AN; alkali-hydrolysis method), available phosphorus (AP; bicarbonate extraction and spectrophotometry method) and available potassium (AK; ammonium acetate extraction and flame photometry method). Soil enzyme activities assessed included catalase (CAT), sucrase (SC), urease (UE) and acid phosphatase (ACP), following the international soil enzyme assay standards using spectrophotometric quantification.

### Soil DNA extraction and microbial community sequencing

Total DNA was extracted from rhizosphere soils using the FastDNA Spin Kit (MP Biomedicals). DNA concentration and integrity were verified using a NanoDrop 2000 spectrophotometer and agarose gel electrophoresis. The V3–V4 region of the bacterial 16S rRNA gene was amplified using primers 338F/806R via PCR. Amplicons were purified and sequenced using the Illumina MiSeq platform (paired-end reads). Raw paired-end reads were processed using the DADA2 plugin in QIIME2 for quality filtering, denoising, merging, and chimera removal, generating high-resolution ASVs. Taxonomic classification was conducted using the classify-sklearn method with a Naive Bayes classifier pre-trained on the SILVA (version 138.2, https://www.arb-silva.de/) database. The default confidence threshold (0.7) was applied, and sequences were assigned to the lowest reliable taxonomic rank; those without sufficient support were labeled as unassigned. Downstream analyses included α-diversity and β-diversity (PCoA, Bray–Curtis distance), microbial co-occurrence network analysis, and random-forest modeling.

### Construction of microbial co-occurrence networks and keystone Taxa identification

Spearman correlation analysis was applied to construct microbial co-occurrence networks based on ASV abundances, using a significance threshold of *p* < 0.05 and correlation coefficient |r| > 0.6. Gephi software was used for network visualization and computation of topological metrics including average degree, clustering coefficient, average path length, and network density. Keystone taxa were identified based on node centrality and network topology features.

### Statistical analysis and structural equation modeling

Statistical analyses were conducted using R and SPSS software. One-way analysis of variance (ANOVA) and Tukey’s HSD test (*p* < 0.05) were used to assess treatment effects. Redundancy analysis (RDA) and Mantel tests were performed to examine correlations between soil properties, enzyme activities, and microbial community composition. Structural equation modeling (SEM) was employed to explore the mechanistic pathways by which fertilization influences sugarcane stem number and yield via soil and microbial variables. Model fit was evaluated using chi-square (χ²), comparative fit index (CFI), and root mean square error of approximation (RMSEA).

## Results

### Combined chemical and organic fertilizers significantly increases sugarcane stem number and yield

According to the analysis of variance results from the Huaqiao and Bankang fields, the FM treatment consistently outperformed other treatments in terms of both sugarcane stem number and yield (**Table 1**). In Huaqiao field, the stem number under the FM treatment reached 7.43×10^4^ stems/ha, which was higher than that of the F treatment (7.28×10^4^ stems/ha) and the CK treatment (6.47×10^4^ stems/ha). Consequently, FM treatment achieved a sugarcane yield of 102.29 t/ha, significantly higher than the F (93.07 t/ha) and CK (70.48 t/ha) treatments. Similarly, in Bankang field, the FM treatment resulted in a stem number of 6.76×10^4^ stems/ha and yield of 123.66 t/ha, highly surpassing both the F (6.22×10^4^ stems/ha and 111.33 t/ha) and CK (5.36×10^4^ stems/ha and 89.04 t/ha) treatments. These results indicate that the combined application of chemical and organic fertilizers significantly increased both sugarcane stem number and yield, outperforming the fertilization with only chemical fertilizer, and demonstrated the importance of integrated fertilization strategy for improving sugarcane productivity.

**Table 1:** Sugarcane stem number and yield under different fertilization treatments in Huaqiao and Bankang fields.

Experimental site	Treatments	Stem number (×10^4^ stems/ha)	Sugarcane yield (t/ha)
Huaqiao field	CK	6.47 ± 0.39a	70.48 ± 9.98c
	F	7.28 ± 0.75a	93.07 ± 3.07b
	FM	7.43 ± 0.58a	102.29 ± 4.39a
Bankang field	CK	5.36 ± 0.36b	89.04 ± 1.13c
	F	6.22 ± 0.63a	111.33 ± 10.23b
	FM	6.76 ± 0.41a	123.60 ± 5.68a

Data are presented as mean ± SD. CK, no fertilizer application; F, only chemical fertilizer application; FM, combined chemical and organic fertilizers application. For each experimental site, the different lowercase letters in the same column indicate significant differences between the treatments at the *p* < 5% level (Tukey’s HSD test).

### Integrated fertilization enhances soil nutrient levels and microbial activity

Significant differences were observed in soil physicochemical properties under three different fertilization treatments in both fields (**Table 2**). Consistently, the pH value was highest under the CK treatment, and significantly reduced under the F and FM treatments; whereas the SOM level showed the best result under the FM treatment at both sites. Regarding nutrients supply, no consistent trends were observed for TN, TP and TK as influenced by different fertilization treatments in both fields, but much higher levels of AN, AP and AK occurred under the F and FM treatments, particularly FM had the highest AK level in both fields. For soil enzyme activities, compared with CK treatment, both F and FM treatments reduced the CAT activity while increasing the ACP activity at the two sites. Activities of SC and UE responded differently under the three fertilization treatments in both fields, but overall FM resulted in relatively high SC and UE activities. In general, the integrated fertilization treatment (FM) improved soil organic matter and available nutrient levels, and maintained relatively high microbial enzyme activity associated with nitrogen (UE) and phosphorus (ACP) cycling.

**Table 2:** Bulk soil properties at the elongation stage of sugarcane under different fertilization treatments in Huaqiao and Bankang fields.

Soil properties	Huaqiao field			Bankang field		
	CK	F	FM	CK	F	FM
pH	4.56 ± 0.12a	4.30 ± 0.10b	4.25 ± 0.08b	4.45 ± 0.14a	4.23 ± 0.18b	4.34 ± 0.17ab
SOM	29.10 ± 5.77ab	26.76 ± 4.72b	30.30 ± 5.05a	37.62 ± 2.74ab	35.96 ± 3.03b	38.86 ± 4.49a
TN	0.15 ± 0.01a	0.16 ± 0.01a	0.16 ± 0.01a	0.16 ± 0.01b	0.16 ± 0.01b	0.18 ± 0.01a
TP	0.05 ± 0.01b	0.06 ± 0.01a	0.05 ± 0.01b	0.05 ± 0.01b	0.08 ± 0.02a	0.07 ± 0.01a
TK	2.64 ± 0.18b	2.74 ± 0.20a	2.90 ± 0.17a	2.49 ± 0.10a	2.64 ± 0.22a	2.20 ± 0.33b
AN	118.63 ± 15.10c	223.76 ± 52.99a	183.99 ± 37.08b	130.27 ± 5.51c	192.55 ± 61.39b	225.07 ± 35.58a
AP	17.56 ± 6.25b	41.27 ± 14.30a	41.16 ± 17.67a	33.90 ± 2.81b	59.42 ± 13.30a	65.97 ± 16.28a
AK	168.57 ± 54.11b	205.61 ± 69.16b	257.59 ± 57.60a	197.63 ± 27.63b	296.06 ± 79.75a	323.20 ± 41.91a
CAT	42.26 ± 7.56a	29.37 ± 5.27b	29.45 ± 8.53b	23.30 ± 6.61a	23.04 ± 2.69a	19.92 ± 2.98b
SC	7.80 ± 1.45a	8.22 ± 1.51a	8.03 ± 1.03a	9.70 ± 2.00a	7.85 ± 1.88b	9.13 ± 1.77ab
UE	329.86 ± 91.50a	261.61 ± 69.74b	321.80 ± 50.38a	317.75 ± 59.49b	360.33 ± 78.90ab	395.56 ± 65.74a
ACP	14.13 ± 4.10b	17.54 ± 2.01a	17.64 ± 3.10a	13.17 ± 3.18b	16.07 ± 3.11a	14.98 ± 3.02ab

Data are presented as mean ± SD. CK, no fertilizer application; F, only chemical fertilizer application; FM, combined chemical and organic fertilizers application. SOM, soil organic matter; TN, total nitrogen; TP, total phosphorus; TK, total potassium; AN, available nitrogen; AP, available phosphorus; AK, available potassium; CAT, catalase; SC, sucrase; UE, urease; ACP, acid phosphatase. For each experimental site, the different lowercase letters in the same row indicate significant differences between the treatments at the *p* < 5% level (Tukey’s HSD test).

### Organic fertilizer enhances the structural differentiation of rhizosphere microbial communities

Based on the taxonomic profiling of 120 soil samples (60 samples from Huaqiao, and 60 samples from Bangkang), *Acidobacteriota* and *Actinobacteriota* were identified as the dominant bacterial phyla across all treatments. Fertilizer application was associated with increased relative abundances of *Proteobacteria* and *Actinobacteriota*, and a decreased abundance of *Acidobacteriota*. These shifts were most pronounced in the FM group, indicating a significant impact of organic fertilizer on rhizosphere microbial composition (**Figures 1B, C**). Alpha diversity analysis showed that microbial richness varied significantly among treatments, with fertilized plots exhibiting higher diversity compared to the control (CK) (**Figure 1D**). Principal coordinate analysis (PCoA) based on Bray–Curtis distances revealed clear structural differentiation of microbial communities among treatments. The FM group was distinctly separated from CK along the first principal coordinate (PCoA1), which explained 22.5% and 20.85% of the total variance in the Huaqiao and Bankang fields, respectively (**Figures 1E, F**). These results suggest that the combined application of chemical and organic fertilizers exerts a stronger effect on microbial community structure than chemical fertilizer alone.

### Root microbiota as biomarkers in three fertilization treatments

**Figure 2:**
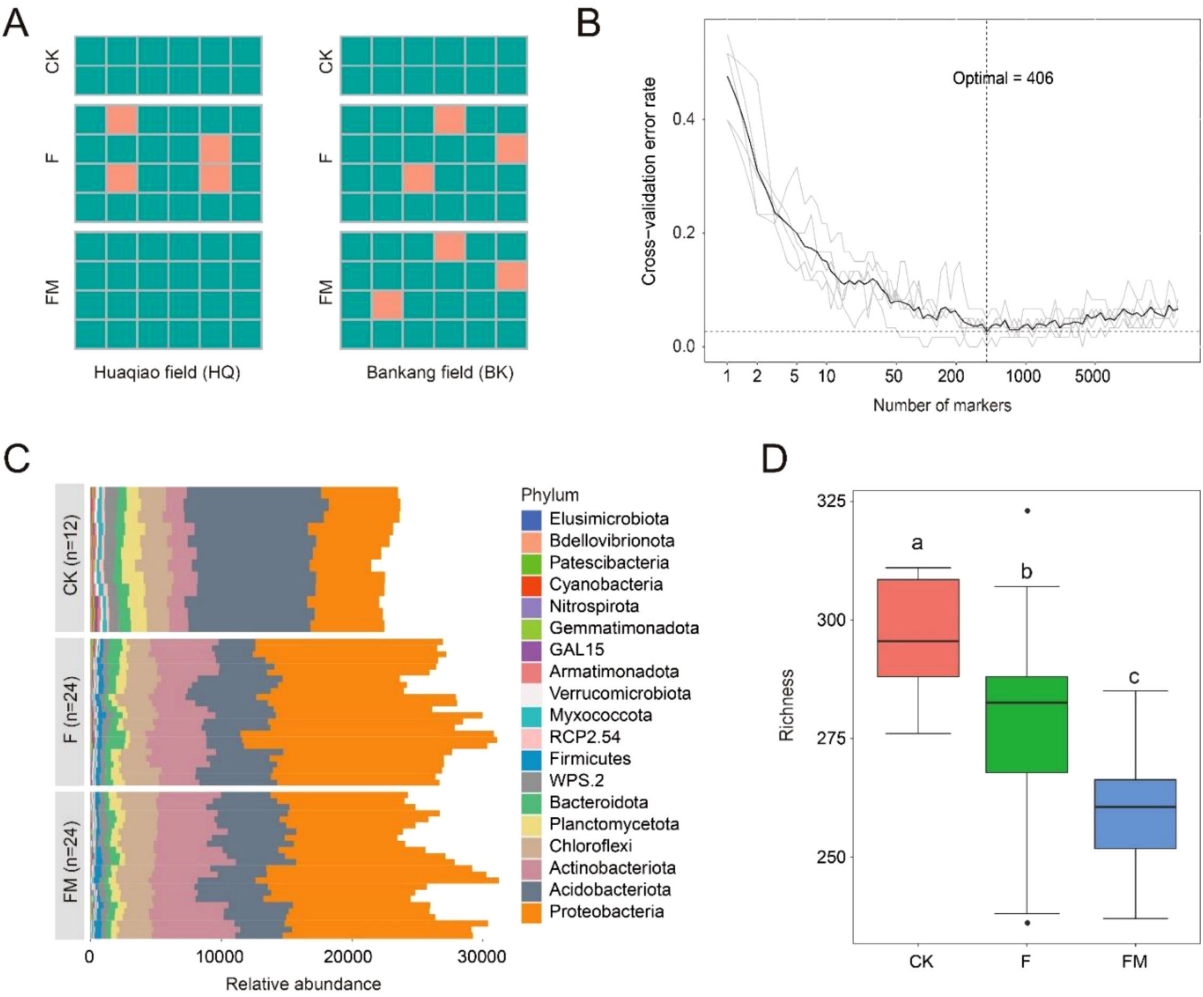
Random-forest model detects bacterial taxa that accurately predict subspeciation under different fertilization treatments. **(A)** The results of the prediction of three different fertilization treatments according to the random-forest model. Each square represents an individual soil sample from 12 CK, 24 F, and 24 F+M replications. The characteristics of the varieties are shown on the left. Accurate predictions are shown in light green, while incorrect predictions are shown in light red. **(B)** The ten-fold cross-validation error as the basis for selecting the optimal number of biomarkers, where 406 signature ASV sequences were selected as the optimal biomarkers. **(C)** The taxonomic information of selected optimal biomarkers and their relative abundance at the phylum level. **(D)** The species diversity index for each fertilization treatment calculated based on the optimal biomarkers, with different lowercase letters representing the differences. CK, no fertilizer application; F, only chemical fertilizer application; FM, combined chemical and organic fertilizers application.

Next, we analyzed whether root microbiota members can be used as biomarkers to differentiate fertilization treatments. We established a model using a random-forest machine-learning method to correlate differentiate fertilization treatments with sugarcane root microbiota data in each site at ASV levels (**Figure 2A**). In relation to fertilizer treatments, the model based on bacterial ASV data showed 93.33% accuracy of root microbiota classification, then we carried out ten-fold cross-validation with five repeats to evaluate the importance of indicator bacterial ASVs. The cross-validation error curve stabilized when the 406 most relevant ASVs were used (**Figure 2B**). Further analysis of the taxonomic composition of these 406 biomarker ASVs at the phylum level revealed that *Proteobacteria* was the dominant phylum across all treatments, with the highest relative abundance observed in the group receiving combined chemical and organic fertilizer application (FM) (**Figure 2C**). This indicates that the addition of organic fertilizer significantly promoted the enrichment of this bacterial group. In addition, the species diversity of these biomarker ASVs was evaluated. The results showed that fertilization, particularly the FM treatment, led to a noticeable reduction in rhizosphere microbial richness (**Figure 2D**). This decline may be attributed to shifts in nutrient input patterns or increased microbial competition within the rhizosphere microenvironment. These results suggest that combined fertilization may exert a suppressive effect on overall microbial diversity, indicating a potential shift toward community consolidation or intensified competition among dominant taxa.

### Combined fertilization reshapes the co-occurrence network structure and enhances the stability of sugarcane rhizosphere microbial communities

**Figure 3:**
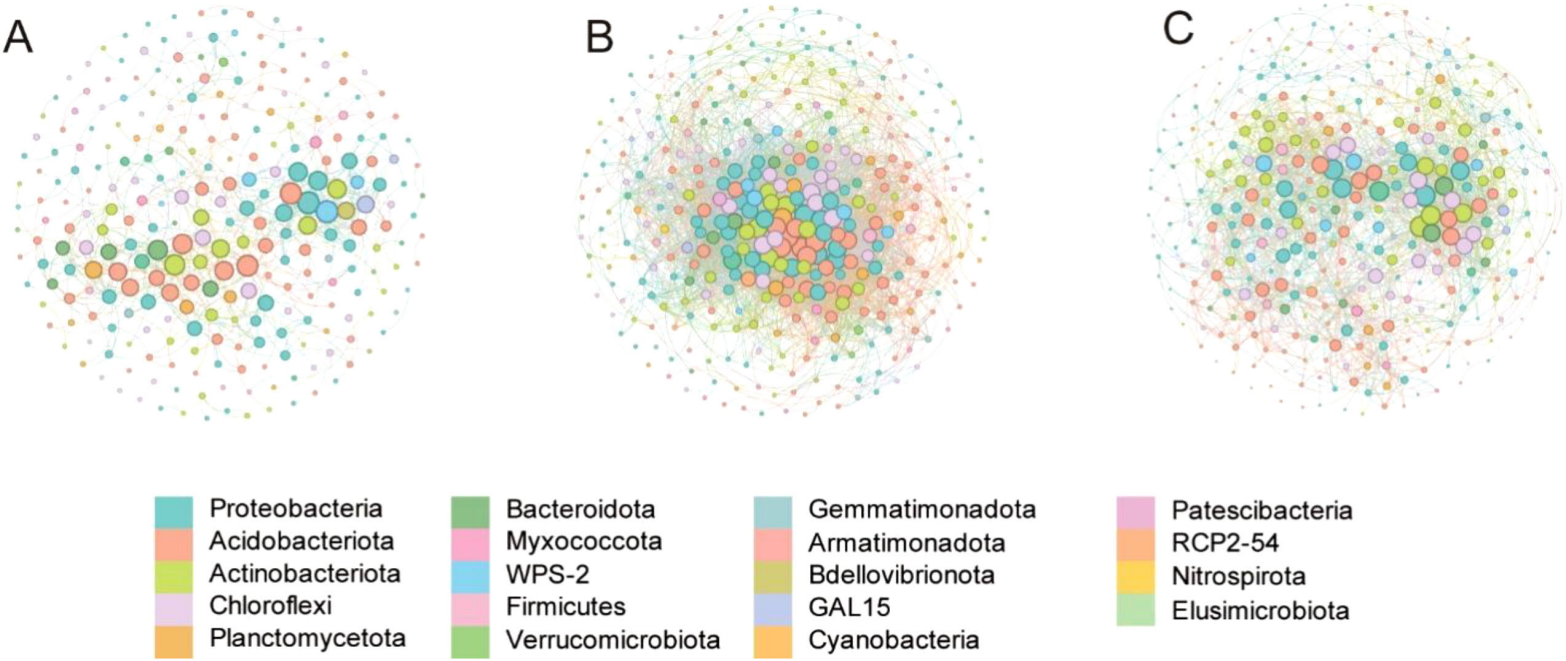
Co-occurrence network of bacterial taxa across three different fertilization conditions. **(A–C)** display networks for CK, F, and FM fertilization condition, respectively, with each taxon color-coded according to its phylum. The legend below the plot indicates the specific taxonomic groups associated with each color. Nodes represent different bacterial taxa, and edges indicate co-occurrence relationships between them. Larger nodes represent more abundant taxa, while smaller nodes represent less abundant ones.

A co-occurrence network of rhizosphere bacterial communities was constructed based on Spearman’s correlation coefficients to evaluate microbial interaction patterns under different fertilization treatments (**Figure 3**). The empirical networks showed characteristic non-random structures, with variations in complexity and connectivity across treatments (**Table 3**). Compared with the control (CK), the solo application of chemical fertilizer (F) greatly increased the number of nodes and edges, resulting in the highest average degree (30.801), shortest average path length (2.933), and increased network density (0.085), indicating intensified microbial interactions. However, this treatment exhibited a lower clustering coefficient (0.231), suggesting a less modular network architecture and potentially reduced local stability. In contrast, the combined application of chemical and organic fertilizers (FM) generated a network with enhanced structural balance. Although the number of connections (edges = 2180) was lower than that of the F treatment, the FM network demonstrated the highest average clustering coefficient (0.522) and the greatest proportion of positive correlations (75.46%), indicating strong cooperative interactions and a more modular and stable network configuration. Moreover, rare bacterial taxa such as *RCP2–54* and *Gemmatimonadota* exhibited increased co-occurrence participation under FM treatment, implying that organic fertilizer may promote ecological integration of low-abundance taxa into the network. Overall, these results suggest that fertilization regimes, particularly the combination of chemical and organic inputs, substantially reshape the rhizosphere microbial co-occurrence network by enhancing cooperative interactions, reinforcing community modularity, and stabilizing microecological structure.

**Table 3:** Co-occurrence networks of keystone bacterial in different fertilization treatments based on correlation analysis.

Treatments	Nodes	Edges	Average degree	Diameter	Density	Mean clustering coefficient	Average path length	Positive	Negative
CK	271	719	5.07	16	0.02	0.43	5.14	63.28	36.72
F	362	5575	30.80	9	0.09	0.23	2.93	59.78	40.22
F+M	344	2180	12.67	9	0.04	0.52	3.46	75.46	24.54

CK, no fertilizer application; F, only chemical fertilizer application; FM, combined chemical and organic fertilizers application.

### Root microbiota biomarkers affect sugarcane yield and stem number

**Figure 4:**
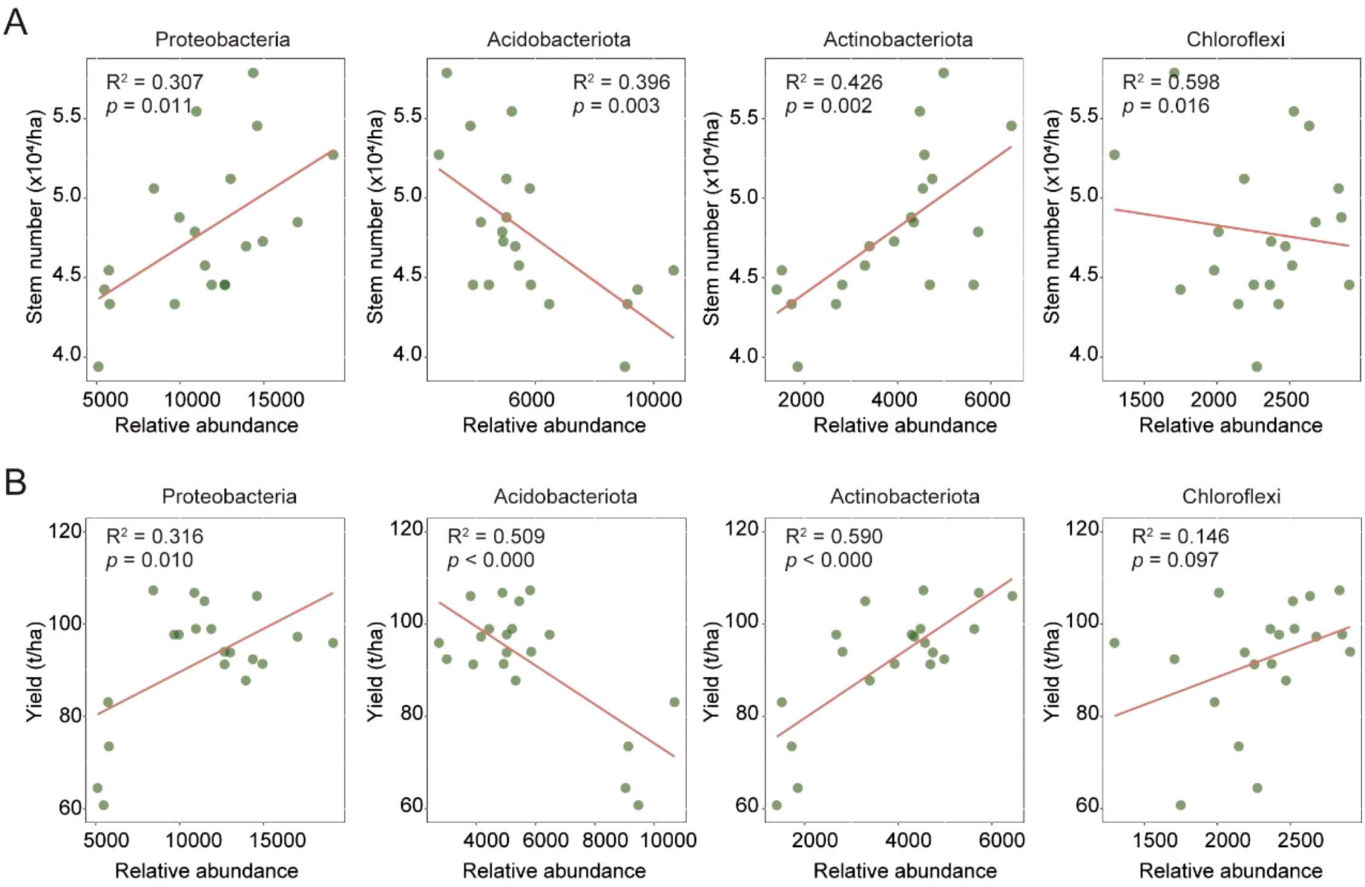
Relationships between the relative abundance of different bacterial phyla and sugarcane stem number **(A)** and yield **(B)**. Each scatter plot displays the linear regression line (red) along with the corresponding R² values and *p*-values, which indicate the strength of the linear relationship and the statistical significance, respectively.

Based on the 406 identified microbial biomarker ASVs, we found that the four most abundant bacterial phyla, i.e., *Actinobacteriota*, *Acidobacteriota*, *Proteobacteria*, and *Chloroflexi*, collectively accounted for over 60% of the total relative abundance, indicating that they represented the dominant components of the biomarker community. To assess their potential functional relevance, we further examined the relationships between the relative abundance of these taxa and key agronomic traits in sugarcane. Significant associations were observed between the dominant microbial phyla and sugarcane yield and stem number (**Figure 4**). Among them, *Actinobacteriota* exhibited positive correlations with both stem number (*p* = 0.426) and yield (*p* = 0.590), suggesting its potential role in promoting sugarcane stem development and productivity. In contrast, *Acidobacteriota* showed a significant negative correlation with stem number (*p* = 0.396) and a similar negative trend with yield (*p* = 0.509), implying that it may be suppressed under high-yield conditions. *Proteobacteria* demonstrated weak positive correlations with both stem number and yield (*p* ≈ 0.3), indicating a limited contribution under current field conditions. *Chloroflexi* displayed a slight negative correlation with stem number (*p* = 0.016) and a low correlation with yield (*p* = 0.146), suggesting minimal involvement in major agronomic outcomes. Taken together, these results highlight *Actinobacteriota* as the most indicative and potentially beneficial taxon among the identified biomarkers, making it a promising target for microbiome-based strategies aimed at improving sugarcane yield and growth performance.

### Soil physicochemical properties, enzyme activities, and microbial communities directly or indirectly promote sugarcane growth

**Figure 5:**
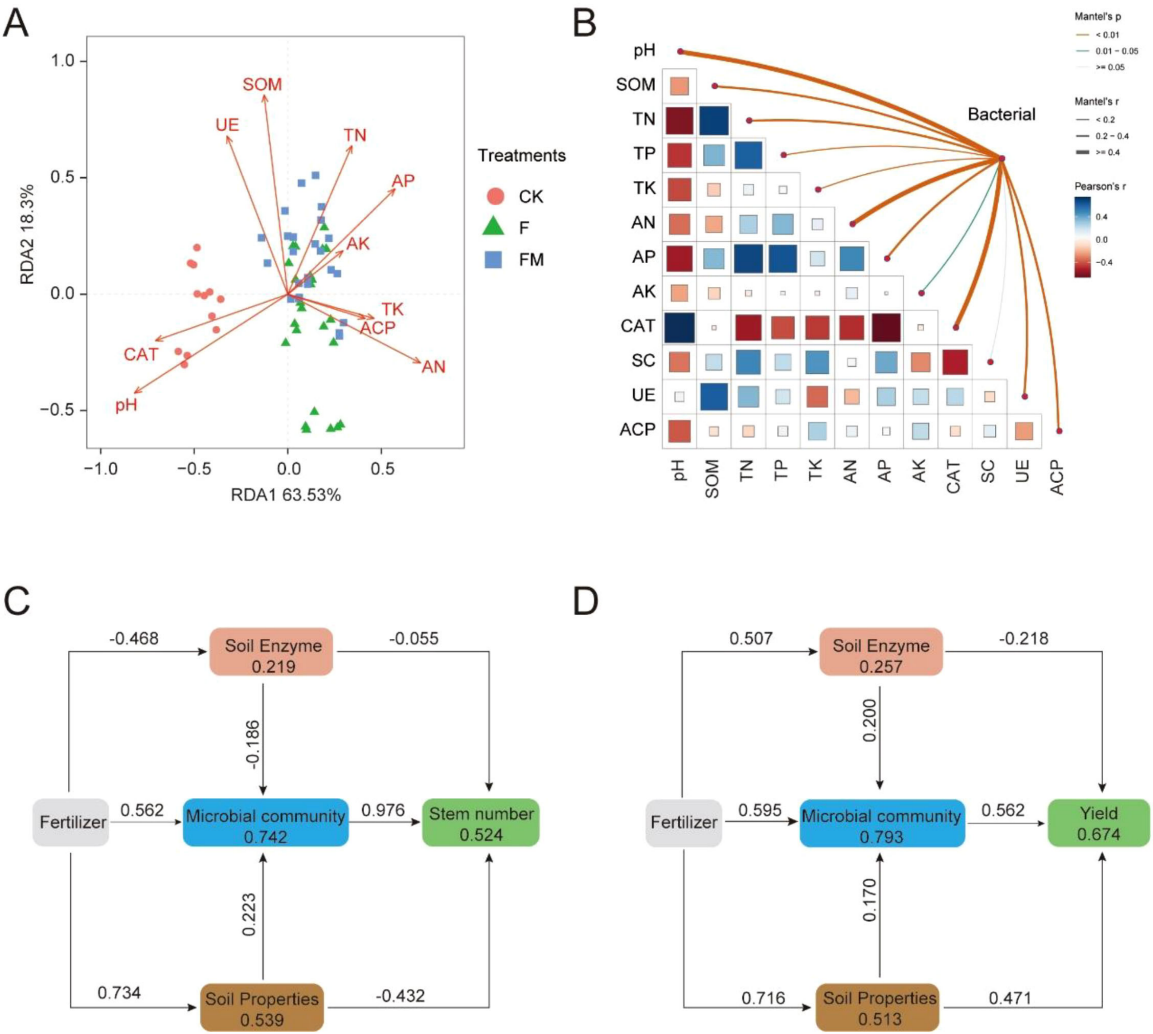
Inference of putative associations among soil physicochemical properties, enzyme activities, microbial communities, and sugarcane yield under different fertilization treatments. **(A)** Redundancy analysis (RDA) visualizing the distribution of soil physicochemical properties across treatments, with arrows representing the contribution of each variable. **(B)** Pearson correlation matrix and Mantel test illustrating the associations between soil properties and microbial communities, with color intensity and line thickness indicating correlation strength and significance levels. **(C, D)** Structural equation modeling (SEM) depicting the direct and indirect effects of fertilization on soil enzyme activity, microbial communities, soil properties with sugarcane stem number and yield, with the number above the arrow indicating relative path coefficient. CK, no fertilizer application; F, only chemical fertilizer application; FM, combined chemical and organic fertilizers application. OM, soil organic matter; TN, total nitrogen; TP, total phosphorus; TK, total potassium; AN, available nitrogen; AP, available phosphorus; AK, available potassium; CAT, catalase; SC, sucrase; UE, urease; ACP, acid phosphatase.

We further explored the relationships among soil physicochemical properties, enzyme activity, microbial community, and crop yield under different treatments. The results of redundancy analysis (RDA) revealed significant differences in soil physicochemical properties across RDA1 (63.53%) and RDA2 (18.3%) axes among the treatment groups (**Figure 5A**). The sample points of the CK group (red circles), F group (green triangles), and FM group (blue squares) exhibited distinct distributions. Soil organic matter (SOM), total nitrogen (TN), available phosphorus (AP), and available potassium (AK) had strong positive effects on microbial community structure, whereas pH and catalase (CAT) showed negative correlations with microbial communities. Pearson correlation matrix and Mantel test results indicated significant associations among soil physicochemical properties, enzyme activity, and microbial communities (**Figure 5B**). Specifically, SOM, AK, and AP were highly correlated with microbial communities (Mantel r > 0.4, *p* < 0.05). The structural equation modeling (SEM) was used to elucidated the pathways through which fertilization affected soil enzyme activity, microbial communities, and sugarcane stem number (**Figure 5C**). The results indicated that microbial communities served as key nodes in regulating stem number (path coefficient = 0.976), while the influence of enzyme activity was relatively weaker (path coefficient = -0.055). Further, the SEM results related to sugarcane yield revealed that fertilization directly enhanced microbial communities (path coefficient = 0.595), while microbial communities exerted significant positive impacts on soil enzyme activity and final yield (path coefficients of 0.200 and 0.562, respectively) (**Figure 5D**). Additionally, soil physicochemical properties demonstrated a notable positive regulatory effect on microbial communities (path coefficient = 0.716). These findings suggest that fertilization not only directly regulates soil microbial communities but also indirectly enhances sugarcane stem number and yield by improving soil enzyme activity and optimizing soil physicochemical properties.

## Discussion

Soil microorganisms play a central role in regulating nutrient cycling, soil fertility, and ecosystem sustainability. Understanding how fertilization strategies influence rhizosphere microbial communities is therefore essential for developing resilient and environmentally friendly cropping systems. In this context, our study provides important insights into how integrated organic and chemical fertilization affects the structure and function of soil microbiota in sugarcane fields, contributing to the broader scientific understanding of microbe-mediated nutrient processes in agroecosystems. This study elucidates the pronounced influence of combined organic and chemical fertilization (FM treatment) on the structural dynamics of the rhizosphere microbiota in sugarcane across Huaqiao and Bankang fields (**Figures 1B, C**). Notably, the FM treatment induced substantial shifts in microbial community composition, particularly in dominant bacterial phyla, including *Acidobacteriota* and *Actinobacteriota*. These findings underscore the capacity of organic fertilizer to modulate microbial assemblages (Gu et al., 2019; Tian et al., 2022), with potential implications for nutrient cycling and plant-microbe interactions. Under FM treatment, the relative abundances of *Proteobacteria* and *Actinobacteriota* markedly increased, while *Acidobacteriota* declined. This reconfiguration likely reflects the introduction of labile carbon sources from organic fertilizer (Tian et al., 2022; Zhang et al., 2021), fostering the proliferation of copiotroph taxa. PCoA analysis further demonstrated (**Figures 1E, F**) distinct segregation of FM-treated communities from those under CK treatment along the primary axis, signifying a robust microbial response to nutrient amendments. Moreover, FM treatment not only increased the abundance of specific taxa but also promoted structural reintegration within the rhizosphere microbiome, organic fertilizers screen out core groups with more functional advantages and closer cooperation, and eliminate those who are not suitable, thus achieving the stability of function and network while the richness is slightly reduced. In our previous study, we found that fertilization directs the selection of soil microorganisms in sugarcane fields (Zhang et al., 2025). Compared to the current study, we suggest that organic fertilizer promotes the selection of specific taxa, enriching beneficial groups while eliminating unsuitable species. This may reduce core taxa diversity but enhance cooperation and niche complementarity among key groups, resulting in synergistic effects on community function and network structure. This may result from enhanced resource availability and niche expansion, facilitating ecological interactions among microbial taxa (Liu et al., 2021b). Given the pivotal role of root-associated microorganisms in mediating nutrient acquisition and plant health (Trivedi et al., 2020), these findings suggest that organic fertilizer exerts a profound restructuring effect on the rhizosphere microbiota, potentially augmenting microbial functional capacity in the agroecosystem.

The application of organic fertilizer was further associated with substantial alterations in the structural integrity and complexity of rhizosphere microbial networks (Gu et al., 2019). Under FM treatment (**Figure 3C**), co-occurrence network analysis revealed a pronounced increase in clustering coefficient and a higher proportion of positive correlations, indicating stronger microbial cooperation and enhanced structural stability. In contrast, F treatment exhibited a higher number of nodes and network density but a relatively low clustering coefficient, suggesting a less modular and potentially less resilient microbial network. This discrepancy may be attributed to the differential effects of organic inputs on microbial community assembly (Jiao et al., 2020), wherein FM treatment fosters synergistic interactions among microbial taxa (Sarsan et al., 2021), thereby reinforcing community stability (Santillan et al., 2025). Additionally, the FM treatment facilitated the integration of rare taxa, such as *RCP2–54* and *Gemmatimonadota*, into the microbial network (Zhao et al., 2022). The incorporation of these low-abundance taxa suggests that organic amendments may mitigate competitive exclusion, enabling the coexistence of diverse microbial populations (Shu et al., 2022). Enhanced microbial network stability under FM treatment aligns with previous reports indicating that increased resource heterogeneity promotes community resilience against environmental perturbations (Hernandez et al., 2021). Collectively, these observations suggest that organic fertilizer, through its impact on microbial community structure and network connectivity, may serve as a critical determinant of microbial stability and functional diversity in the rhizosphere (Li et al., 2024; Shi et al., 2024).

The FM treatment significantly augmented sugarcane stem number and yield across both study sites, indicating that the integration of organic fertilizer is a viable strategy for improving crop productivity (**Table 1**). Mechanistically, this enhancement can be attributed to both direct and indirect pathways involving nutrient bioavailability, microbial activity, and rhizosphere microenvironment optimization (Liu et al., 2022; Zhou et al., 2025). First, FM treatment improved key soil physicochemical properties, notably organic matter and available nutrients supply (**Table 2**). These parameters are critical for nutrient acquisition and plant growth (Han et al., 2021; Ma et al., 2025), although the available nitrogen (AN) content under the FM treatment was sometimes lower than that under the F treatment, the yield remained the highest. This highlights the key role of organic fertilizer-driven microbial pathways in supporting plant growth, offering benefits beyond the immediate supply of individual nutrients. We speculate that the improved rhizosphere microbial community under organic fertilization promotes crop growth through multiple mechanisms, compensating for, and even surpassing, the short-term effects of chemical nitrogen fertilizer alone. In particular, the observed increase in available phosphorus and potassium under FM treatment may have facilitated nutrient uptake (Cao et al., 2012; Li et al., 2023), thereby sustaining higher biomass accumulation in sugarcane (Zhao et al., 2020). Second, the FM treatment induced pronounced changes in rhizosphere microbial community structure, characterized by increased relative abundances of *Actinobacteriota* and *Proteobacteria* (Rodríguez-Caballero et al., 2017). These microbial taxa are known to play vital roles in organic matter decomposition, nutrient mineralization, and plant growth promotion (Hartmann and Six, 2023). According to the previous study, *Actinobacteriota produce IAA, cytokinins, and gibberellins, which are crucial for plant root development, nutrient uptake, and enhancing soil nutrient availability* (Waadt et al., 2022). *In this study, we suggest that the enrichment of these bacteria in the sugarcane rhizosphere significantly contributes to yield.*

SEM analysis indicated that microbial activity under FM treatment exerted significant positive effects on sugarcane stem number and yield (**Figures 5C, D**), probably mediated through enhanced nitrogen and phosphorus cycling enzyme activity (**Table 3**) and organic matter mineralization (Xia et al., 2025). Furthermore, the FM treatment promoted the establishment of cooperative microbial networks, characterized by higher clustering coefficients and increased positive correlations among microbial taxa (Hernandez et al., 2021; Meng et al., 2022). This structural reorganization likely facilitated nutrient exchange and resource partitioning within the microbial community, contributing to improved plant nutrient acquisition and growth performance (Cao et al., 2024). These findings underscore the multifaceted mechanisms through which organic fertilizer enhances sugarcane yield, encompassing nutrient enrichment, microbial community restructuring, and the reinforcement of cooperative interactions within the rhizosphere microbiome, by improving soil enzyme activity and optimizing soil physicochemical properties (Afridi et al., 2022), while fungi and other eukaryotic microorganisms, which play important roles in shaping soil ecosystem functions, were not examined in this study, they will be the focus of further investigation. In addition, long-term microbial dynamics, ecological feedbacks, and variations across different environmental or geographical contexts will also be addressed in future studies.

## Conclusion

This study demonstrates that the combined application of chemical and organic fertilizers exerts a pronounced influence on the structure and functional capacity of the sugarcane rhizosphere microbiome, with significant implications for crop yield and soil health. FM treatment enhanced microbial network complexity and stability, promoting cooperative interactions and more efficient resource exchange within the rhizosphere. Accordingly, it strengthened the links between the rhizosphere microbial community, soil properties, enzyme activity, and final crop performance, thereby directly or indirectly increasing sugarcane stem number and yield, and improving soil nutrient availability, enzyme activity, and microbial network connectivity. These findings suggest that the integration of organic fertilizer into conventional chemical fertilization regime represents a promising strategy for promoting sustainable sugarcane production through the modulation of rhizosphere microbial ecology.

## Data Availability

The data presented in the study are deposited in the NCBI repository, accession number PRJNA1401410.

## References

[B1] Afridi M. S. Fakhar A. Kumar A. Ali S. Medeiros F. H. V. Muneer M. A. . (2022). Harnessing microbial multitrophic interactions for rhizosphere microbiome engineering. Microbiol. Res. 265, 127199. doi: 10.1016/j.micres.2022.127199, PMID: 36137486

[B2] Cao N. Chen X. Cui Z. Zhang F. (2012). Change in soil available phosphorus in relation to the phosphorus budget in China. Nutrient Cycling Agroecosyst 94, 161–170. doi: 10.1007/s10705-012-9530-0

[B3] Cao T. Luo Y. Shi M. Tian X. Kuzyakov Y. (2024). Microbial interactions for nutrient acquisition in soil: Miners, scavengers, and carriers. Soil Biol. Biochem. 188, 109215. doi: 10.1016/j.soilbio.2023.109215

[B4] Chen Y. Tu P. Yang Y. Xue X. Feng Z. Dan C. . (2022). Diversity of rice rhizosphere microorganisms under different fertilization modes of slow-release fertilizer. Sci. Rep. 12, 2694. doi: 10.1038/s41598-022-06155-1, PMID: 35177664 PMC8854673

[B5] Cui X. Zhang Y. Gao J. Peng F. Gao P. (2018). Long-term combined application of manure and chemical fertilizer sustained higher nutrient status and rhizospheric bacterial diversity in reddish paddy soil of Central South China. Sci. Rep. 8, 16554. doi: 10.1038/s41598-018-34685-0, PMID: 30410029 PMC6224536

[B6] Dang P. Li C. Lu C. Zhang M. Huang T. Wan C. . (2022). Effect of fertilizer management on the soil bacterial community in agroecosystems across the globe. Agric Ecosyst. Environ. 326, 107795. doi: 10.1016/j.agee.2021.107795

[B7] Gu S. Hu Q. Cheng Y. Bai L. Liu Z. Xiao W. . (2019). Application of organic fertilizer improves microbial community diversity and alters microbial network structure in tea (Camellia sinensis) plantation soils. Soil Tillage Res. 195, 104356. doi: 10.1016/j.still.2019.104356

[B8] Guan Q. Tang J. Davis K. F. Kong M. Feng L. Shi K. . (2025). Improving future agricultural sustainability by optimizing crop distributions in China. PNAS nexus 4, pgae562. doi: 10.5194/egusphere-egu25-21668, PMID: 39777291 PMC11705388

[B9] Han J. Dong Y. Zhang M. (2021). Chemical fertilizer reduction with organic fertilizer effectively improve soil fertility and microbial community from newly cultivated land in the Loess Plateau of China. Appl. Soil Ecol. 165, 103966. doi: 10.1016/j.apsoil.2021.103966

[B10] Hartmann M. Six J. (2023). Soil structure and microbiome functions in agroecosystems. Nat. Rev. Earth Environ. 4, 4–18. doi: 10.1038/s43017-022-00366-w

[B11] Hernandez D. J. David A. S. Menges E. S. Searcy C. A. Afkhami M. E. (2021). Environmental stress destabilizes microbial networks. ISME J. 15, 1722–1734. doi: 10.1038/s41396-020-00882-x, PMID: 33452480 PMC8163744

[B12] Hossain A. Krupnik T. J. Timsina J. Mahboob M. G. Chaki A. K. Farooq M. . (2020). “ Agricultural land degradation: processes and problems undermining future food security,” in Environment, climate, plant and vegetation growth. Eds. Fahad S. Hasanuzzaman M. Alam M. Ullah H. Saeed M. Ali Khan I. Adnan M. ( Springer International Publishing, Cham), 17–61.

[B13] Jiao S. Yang Y. Xu Y. Zhang J. Lu Y. (2020). Balance between community assembly processes mediates species coexistence in agricultural soil microbiomes across eastern China. ISME J. 14, 202–216. doi: 10.1038/s41396-019-0522-9, PMID: 31611655 PMC6908645

[B14] Li H. Hu Y. Liu G. Sheng J. Zhang W. Zhao H. . (2023). Responses of biomass accumulation and nutrient utilization along a phosphorus supply gradient in Leymus chinensis. Sci. Rep. 13, 5660. doi: 10.1038/s41598-023-31402-4, PMID: 37024558 PMC10079846

[B15] Li K. Xing X. Wang S. Liao R. Hassan M. U. Aamer M. . (2024). Organic fertilization enhances network complexity among bacteria, fungi, and protists by improving organic matter and phosphorus in acidic agricultural soils. Eur. J. Soil Biol. 122, 103649. doi: 10.1016/j.ejsobi.2024.103649

[B16] Li X. Xiao R. Tao Y. (2025). Enhancing plant stress resilience and agricultural sustainability through rhizosphere microbiome optimization. Plant Soil. doi: 10.1007/s11104-025-07359-w

[B17] Liu S. He F. Kuzyakov Y. Xiao H. Hoang D. T. T. Pu S. . (2022). Nutrients in the rhizosphere: A meta-analysis of content, availability, and influencing factors. Sci. Tot Environ. 826, 153908. doi: 10.1016/j.scitotenv.2022.153908, PMID: 35183641

[B18] Liu J. Li S. Yue S. Tian J. Chen H. Jiang H. . (2021b). Soil microbial community and network changes after long-term use of plastic mulch and nitrogen fertilization on semiarid farmland. Geoderma 396, 115086. doi: 10.1016/j.geoderma.2021.115086

[B19] Liu B. Wang X. Ma L. Chadwick D. Chen X. (2021a). Combined applications of organic and synthetic nitrogen fertilizers for improving crop yield and reducing reactive nitrogen losses from China’s vegetable systems: A meta-analysis. Environ. pollut. 269, 116143. doi: 10.1016/j.envpol.2020.116143, PMID: 33310496

[B20] Luo C. He Y. Chen Y. (2025). Rhizosphere microbiome regulation: Unlocking the potential for plant growth. Curr. Res. Microbial Sci. 8, 100322. doi: 10.1016/j.crmicr.2024.100322, PMID: 39678067 PMC11638623

[B21] Ma X. Ma W. Wang C. Xu Y. (2025). Nitrogen and phosphorus supply controls stability of soil organic carbon in alpine meadow of the Qinghai-Tibetan Plateau. Agric Ecosyst. Environ. 379, 109336. doi: 10.1016/j.agee.2024.109336

[B22] Meng L. Xu C. Wu F. Huhe (2022). Microbial co-occurrence networks driven by low-abundance microbial taxa during composting dominate lignocellulose degradation. Sci. Tot Environ. 845, 157197. doi: 10.1016/j.scitotenv.2022.157197, PMID: 35839876

[B23] Philippot L. Chenu C. Kappler A. Rillig M. C. Fierer N. (2024). The interplay between microbial communities and soil properties. Nat. Rev. Microbiol. 22, 226–239. doi: 10.1038/s41579-023-00980-5, PMID: 37863969

[B24] Rodríguez-Caballero G. Caravaca F. Fernández-González A. J. Alguacil M. M. Fernández-López M. Roldán A. . (2017). Arbuscular mycorrhizal fungi inoculation mediated changes in rhizosphere bacterial community structure while promoting revegetation in a semiarid ecosystem. Sci. Tot Environ. 584-585, 838–848. doi: 10.1016/j.scitotenv.2017.01.128, PMID: 28131451

[B25] Santillan E. Neshat S. A. Wuertz S. (2025). Disturbance and stability dynamics in microbial communities for environmental biotechnology applications. Curr. Opin. Biotechnol. 93, 103304. doi: 10.1016/j.copbio.2025.103304, PMID: 40245612

[B26] Sarsan S. Pandiyan A. Rodhe A. V. Jagavati S. (2021). “ Synergistic interactions among microbial communities,” in Microbes in microbial communities: ecological and applied perspectives. Eds. Singh R. P. Manchanda G. Bhattacharjee K. Panosyan H. ( Springer Singapore, Singapore), 1–37.

[B27] Shi T.-S. Collins S. L. Yu K. Peñuelas J. Sardans J. Li H. . (2024). A global meta-analysis on the effects of organic and inorganic fertilization on grasslands and croplands. Nat. Commun. 15, 3411. doi: 10.1038/s41467-024-47829-w, PMID: 38649721 PMC11035549

[B28] Shi Z. Ma L. Wang X. Wu S. Bai J. Li Z. . (2023). Efficiency of agricultural modernization in China: Systematic analysis in the new framework of multidimensional security. J. Clean Prod 432, 139611. doi: 10.1016/j.jclepro.2023.139611

[B29] Shu X. He J. Zhou Z. Xia L. Hu Y. Zhang Y. . (2022). Organic amendments enhance soil microbial diversity, microbial functionality and crop yields: A meta-analysis. Sci. Tot Environ. 829, 154627. doi: 10.1016/j.scitotenv.2022.154627, PMID: 35306065

[B30] Tian S. Zhu B. Yin R. Wang M. Jiang Y. Zhang C. . (2022). Organic fertilization promotes crop productivity through changes in soil aggregation. Soil Biol. Biochem. 165, 108533. doi: 10.1016/j.soilbio.2021.108533

[B31] Tripathi S. Srivastava P. Devi R. S. Bhadouria R. (2020). “ Chapter 2 - Influence of synthetic fertilizers and pesticides on soil health and soil microbiology,” in Agrochemicals detection, treatment and remediation. Ed. Prasad M. N. V. ( Butterworth-Heinemann), 25–54.

[B32] Trivedi P. Leach J. E. Tringe S. G. Sa T. Singh B. K. (2020). Plant–microbiome interactions: from community assembly to plant health. Nat. Rev. Microbiol. 18, 607–621. doi: 10.1038/s41579-020-0412-1, PMID: 32788714

[B33] Waadt R. Seller C. A. Hsu P. K. Takahashi Y. Munemasa S. Schroeder J. I. (2016). Plant hormone regulation of abiotic stress responses. Nat. Rev. Mol. Cell Biol. 23, 680–694. doi: 10.1038/s41580-022-00479-6, PMID: 35513717 PMC9592120

[B34] Wei W. Yan Y. Cao J. Christie P. Zhang F. Fan M. (2016). Effects of combined application of organic amendments and fertilizers on crop yield and soil organic matter: An integrated analysis of long-term experiments. Agric Ecosyst. Environ. 225, 86–92. doi: 10.1016/j.agee.2016.04.004

[B35] Xia M. Li P. Liu J. Qin W. Dai Q. Wu M. . (2025). Long-term fertilization promotes the microbial-mediated transformation of soil dissolved organic matter. Commun. Earth Environ. 6, 114. doi: 10.1038/s43247-025-02032-7

[B36] Xie W. Zhu A. Ali T. Zhang Z. Chen X. Wu F. . (2023). Crop switching can enhance environmental sustainability and farmer incomes in China. Nature 616, 300–305. doi: 10.1038/s41586-023-05799-x, PMID: 36927804

[B37] Yang J. Liu X. Rong X. Jiang P. Xia Y. Xie G. . (2025). Bio-organic fertilizer application improves cucumber growth, disease resistance, and soil fertility by regulating rhizosphere microbiomes. Plant Soil. doi: 10.1007/s11104-025-07460-0

[B38] Yousaf M. Li J. Lu J. Ren T. Cong R. Fahad S. . (2017). Effects of fertilization on crop production and nutrient-supplying capacity under rice-oilseed rape rotation system. Sci. Rep. 7, 1270. doi: 10.1038/s41598-017-01412-0, PMID: 28455510 PMC5430767

[B39] Yu Y. Zhang Q. Kang J. Xu N. Zhang Z. Deng Y. . (2024). Effects of organic fertilizers on plant growth and the rhizosphere microbiome. Appl. Environ. Microbiol. 90, e01719–e01723. doi: 10.1128/aem.01719-23, PMID: 38193672 PMC10880660

[B40] Zhang Z. Yan J. Han X. Zou W. Chen X. Lu X. . (2021). Labile organic carbon fractions drive soil microbial communities after long-term fertilization. Global Ecol. Conserv. 32, e01867. doi: 10.1016/j.gecco.2021.e01867

[B41] Zhao Z. Ma Y. Feng T. Kong X. Wang Z. Zheng W. . (2022). Assembly processes of abundant and rare microbial communities in orchard soil under a cover crop at different periods. Geoderma 406, 115543. doi: 10.1016/j.geoderma.2021.115543

[B42] Zhao D. Momotaz A. LaBorde C. Irey M. (2020). Biomass yield and carbohydrate composition in sugarcane and energy cane grown on mineral soils. Sugar Tech 22, 630–640. doi: 10.1007/s12355-020-00807-0

[B43] Zhou D. Li S. Yu P. Xiu N. Zhao Y. Dong Q. . (2025). Microbial mechanisms underlying complementary soil nutrient utilization regulated by maize-peanut root exudate interactions. Rhizosphere 33, 101051. doi: 10.1016/j.rhisph.2025.101051

[B44] Zhang Z. Wang Y. Ai J. Dao J. Li A. Deng J. . (2025). Effects of potassium fertilizer on rhizosphere microbial diversity and community assembly in sugarcane. Chinese Journal of Applied Ecology, 36, 526–536. doi: 10.13287/j.1001-9332.202501.015, PMID: 40370170

